# Effect of 940 nm diode laser adjunct to mechanical debridement on S100A8 levels and clinical parameters in peri-implantitis treatment

**DOI:** 10.1007/s10103-026-04925-1

**Published:** 2026-06-24

**Authors:** Akif Nalbant, Hatice Ebru Olgun, Meltem Hendek, Osman Çağlayan

**Affiliations:** 1 Department of Periodontology, Kırıkkale University, Kırıkkale, Turkey; 2 Department of Medical Biochemistry, Kırıkkale University, Kırıkkale, Turkey

**Keywords:** Biomarkers, Calprotectin, Inflammation, Laser therapy, Peri-implantitis

## Abstract

The aim of this study was to compare the effectiveness of diode laser and combined treatment protocols, applied in addition to mechanical debridement, in the treatment of peri-implantitis on clinical periodontal parameters and S100A8 levels in peri-implant sulcus fluid (PISF). A total of 39 patients diagnosed with peri-implantitis were divided into three groups based on the treatment protocols used: Mechanical Debridement, Diode Laser, and Combined Treatment. Probing depth, clinical attachment level, modified plaque index, gingival index, and PISF volume measurements were recorded at baseline and at weeks 4 and 12. S100A8 levels in PISF samples were measured by ELISA. During the 12-week follow-up period, statistically significant improvements in clinical parameters were observed in all groups compared with baseline (p < 0.05). In the intergroup comparison, the most significant decrease and statistical superiority in mPI and GI values were observed in the combined treatment group (p < 0.001). Although there was no statistically significant difference among the groups in probing depth and clinical attachment gain, the greatest numerical improvement was observed in the combined group. Despite clinical improvement, no significant change in total S100A8 levels was detected between groups or over time (p > 0.05). The combined use of mechanical debridement and a diode laser in the treatment of peri-implantitis is more effective at reducing clinical signs of inflammation than either method used alone. However, the lack of correlation between S100A8 levels and clinical improvement suggests that this biomarker may not be sensitive in short-term follow-up of treatment.

## Introduction

Dental implant treatment is one of the most frequently applied treatment options today. With the increasing number of dental implant applications, peri-implant diseases and biomechanical complications are also increasing. Peri-implant mucositis and peri-implantitis are the most common peri-implant diseases [1]. The prevalence of peri-implant diseases is increasing over time [2]. In a recent meta-analysis, the prevalence of peri-implantitis was reported as 12.53% at the implant level and 19.53% at the patient level [3].

The main goal of peri-implantitis treatment is to stop the infection and restore tissue health. The primary method for this is non-surgical mechanical debridement. However, the threaded structure and rough surfaces of implants make mechanical debridement difficult. Due to these limitations, chemical agents or laser systems have been suggested in addition to mechanical debridement. Lasers are promising due to their bactericidal effects and their ability to stimulate tissue healing [4]. Due to this limitation, approaches such as chemical agents, air-powder systems for surface decontamination, and rotary brushes have been suggested in addition to mechanical debridement [5]. Laser treatments, in particular, are considered promising due to their potential to reduce biofilm and microbial load, reduce tissue inflammation, and improve clinical parameters such as probing depth and bleeding on probing [6]. However, systematic evaluations show that the consistent superiority of lasers or other methods in preventing bone loss or long-term damage to the implant surface remains unclear. Therefore, treatment protocols may include the use of various laser systems in addition to or as an alternative to mechanical debridement alone [7]. The success of peri-implantitis treatment is traditionally evaluated using clinical and radiographic parameters such as probing depth, bleeding on probing, and attachment loss; however, these values may reflect past tissue destruction rather than the molecular level of active inflammation [8]. In addition, clinical measurements may vary among practitioners and may not be sensitive to detecting early inflammatory changes [9]. In this context, peri-implant sulcus fluid (PISF) biomarkers have been frequently investigated in the literature due to their potential to reflect immunological and inflammatory processes in peri-implant tissues earlier and more accurately [10, 11]. Although S100A8 is reported in the literature as a pro-inflammatory cytokine, it also exhibits anti-inflammatory properties under specific conditions, helping prevent tissue damage caused by intense inflammation [12]. S100A8 levels have been used as an inflammation marker in oral fluids and have decreased in saliva samples before and after peri-implant disease treatment. However, in this study, the authors did not find a significant correlation between saliva and PISF levels [13].

The aim of this study is to evaluate the effectiveness of mechanical debridement, diode laser therapy, and combined mechanical debridement and diode laser therapy in the treatment of peri-implantitis on clinical parameters and S100A8 levels in PISF.

The null hypothesis tested was that the adjunctive use of a 940 nm diode laser would provide no additional benefit in terms of clinical parameters and PISF S100A8 levels compared to mechanical debridement alone.

## Materials and methods

This study was conducted in full compliance with the principles of the Helsinki Declaration, and the study protocol was approved by the Research Ethics Committee of the Kirikkale University (Decision No: 2024.12.21). The study, conducted as a prospective, comparative clinical research study, included individuals who applied to the Department of Periodontology at the Faculty of Dentistry. After detailed information about the research purpose and the procedures to be followed was provided, written informed consent forms were obtained from all participants. At the beginning of the study, 45 implants meeting the inclusion criteria were randomized into three study groups, each consisting of 15 implants. Following the samples excluded from analysis due to losses during the follow-up process and technical reasons; Data from a total of 39 implants were included in the final analysis, divided into three groups: Group 1 (*n* = 13) (only mechanical debridement), Group 2 (*n* = 13) (only diode laser monotherapy), and Group 3 (*n* = 13) (combined mechanical debridement and diode laser). The study population consisted of individuals aged 30–65 years. Inclusion criteria were: implants being functional, showing no clinical mobility, and having a probing depth of 6 mm or more. Conversely, individuals with systemic diseases that could affect healing, smokers and alcohol users, pregnant or lactating women, those with a history of radiation/chemotherapy, those with parafunctional habits such as teeth grinding, those who had used systemic antibiotics in the last 6 months, or those who had received peri-implantitis treatment in the relevant area in the last 3 months were excluded from the evaluation. The patients’ full names, ages, genders, contact information, implant numbers used, and pre-treatment clinical status were recorded on the case report form. Patients were assigned to groups according to a simple randomization method. Before treatment, clinical periodontal parameters of all dental implants included in the study, such as probing depth (PD), clinical attachment level (CAL), modified plaque index (mPI) [14], and gingival index (GI) [15], were recorded, and PISF samples were collected. PD and CAL [16] were calculated from 6 sites of each dental implant, and mPI and GI from 4 sites [17].

Before collecting PISF samples, supragingival dental plaque on the relevant peri-implant surface was removed using sterile plastic curettes. The surface was air-dried and isolated with cotton rolls. PISF samples were collected from two areas, mesial and distal, of peri-implantitis-affected dental implants using paper strips (Ora Flow, Inc., Amityville, NY, USA). Standard paper strips were inserted 1 mm into the peri-implant sulcus entrance and held for 30 s. During this procedure, care was taken to minimize mechanical trauma to avoid any increase in PISF volume. Paper strips contaminated with blood or saliva were discarded and excluded from the evaluation. The paper strips were transferred to a pre-calibrated Periotron 8000 device (Oraflow, Inc., Plainview, NY, USA). The microliter (µL) equivalents of the device readings were recorded. PISS samples, placed in sterile Eppendorf tubes, were stored first at -20 °C and then at -80 °C until laboratory analysis. All clinical peri-implant measurements and PISF volume were recorded on the case report form. After recording the baseline clinical peri-implant parameters and collecting PISF samples in all groups, group-specific treatment procedures were applied.

In the mechanical debridement group (Group 1), subgingival mechanical debridement was performed using sterile titanium curettes to remove biofilm and attachments from the implant surface. In the diode laser group (Group 2), decontamination was performed using a diode laser (Epic10, Biolase, CA, USA) with a wavelength of 940 ± 10 nm, a power of 2.5 W, and CP2 (Comfort Pulse 2) mode. A 400 μm-thick fiber tip (E4, 7 & 9 mm) was used during application. The laser tip was inserted into the base of the peri-implant pocket, slightly withdrawn, and then applied along the subgingival implant surface with vertical and horizontal scanning movements for 30 s. In the combined treatment group (Group 3), a sequential treatment protocol was followed. Immediately after the mechanical debridement procedure described in Group 1, diode laser application was performed with the parameters and application time used in Group 2. Following completion of the treatment procedures, peri-implant pockets in all groups were washed with 10 cc of 10% povidone-iodine solution. Clinical parameters measured in implant on day 0 (T0), 4th week (T1) and 12th (T2) were statistically analyzed, and the collected PISF samples were analyzed first biochemically and then statistically.

### Biochemical analysis

S100A8 levels in the stored PISF samples were analyzed using an ELISA kit (Human S100A8 ELISA Kit, YL Biont, Shanghai/China) according to the manufacturer’s instructions. Optical density values were read at a wavelength of 450 nm in a microplate reader, and the results were calculated in ng/mL using a standard curve.

### Statistical analysis

Statistical power analysis of the study was performed using G*Power software; the test power (1-β) of the study with a total of 39 implants was calculated as 85%. Data analysis was performed using IBM SPSS Statistics 27.0. Non-parametric tests were preferred because the sample sizes in each group were *n* < 30. Categorical variables were summarized as numbers and percentages (%), and numerical variables were summarized with median (minimum-maximum) values. The Kruskal-Wallis test was used for comparisons between independent groups, while the Mann-Whitney U test was used for pairwise comparisons to determine the source of the difference. The Friedman test and the Wilcoxon Signed-Rank test were applied to analyze time-dependent changes within groups (T0, T1, T3). A statistical significance level of *p* < 0.05 was accepted.

## Results

### Demographic data

**Fig. 1:**
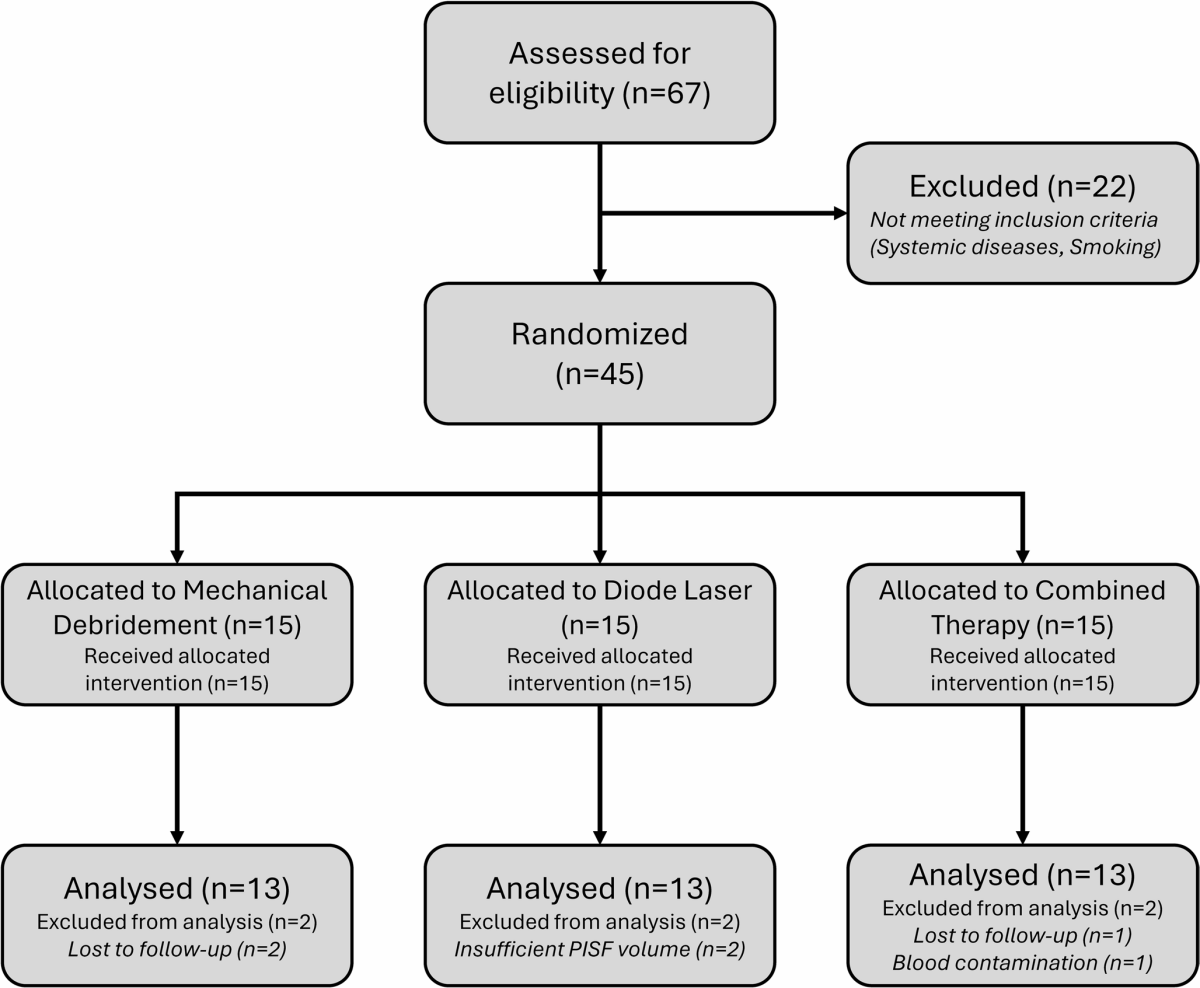
CONSORT flow diagram illustrating the study design, recruitment process, randomization, allocation to treatment groups (Group 1: Mechanical Debridement, Group 2: Diode Laser, Group 3: Combined Therapy), and the final number of implants included in the analysis

Patient selection, randomization, and follow-up process are presented in Fig. 1 according to CONSORT criteria. A total of 67 individuals were initially evaluated for eligibility. After eliminating 22 participants who did not meet the inclusion criteria (due to systemic disease, smoking, etc.), the remaining 45 patients were randomized and distributed into study groups (*n* = 15/group). After samples were excluded due to losses during follow-up and technical issues, a total of 39 patients (*n* = 13 per group) were included in the final statistical analysis. The demographic characteristics of the study groups are shown in Table 1. As a result of the statistical analysis, no significant difference was found between the groups in age or gender distribution (*p* > 0.05), and the groups were found to be homogeneous at the beginning of the study.

**Table 1:** Demographic data in groups

Variable		Groups			*P*-value
		Mechanical Debridement	Diode Laser	Combined	
Gender n (%)	Male	7 (53.8)	6 (46.2)	7 (53.8)	0.902
	Female	6 (46.2)	7 (53.8)	6 (46.2)	
Age	Min-Max (Median)	43.0–61.0 (49.0)	40.0–52.0 (47.0)	45.0–55.0 (49.0)	0.089
	Mean ± SD	50.38 ± 5.42	46.62 ± 3.43	49.62 ± 3.07	

SD: standard deviation

### Changes in clinical and biochemical parameters

The changes in all clinical and biochemical values over time are presented in Tables 2 and 3.

**Table 2:** Changes in clinical parameters over time

	Groups			
Parameters & Time	Mechanical Debridement	Diode Laser	Combined	*P*-value
PD (mm)				
Baseline	5.14 ± 0.60	6.32 ± 0.60^a)^	6.28 ± 0.62^a)^	< 0.05
4th week	4.90 ± 0.66^c)^	5.49 ± 0.67^c)^	5.37 ± 0.60^c)^	0.103
12th week	4.72 ± 0.72^c), d)^	5.36 ± 0.50^c)^	5.04 ± 0.49^c), d)^	0.062
*P-*value	0.000	0.000	0.000	
CAL (mm)				
Baseline	3.88 ± 0.88	4.62 ± 0.61^a)^	4.08 ± 0.60^b)^	0.037
4th week	3.62 ± 0.88^c)^	4.49 ± 0.64^a)^, ^c)^	3.78 ± 0.58^b)^, ^c)^	0.010
12th week	3.54 ± 0.83^c), d)^	4.18 ± 0.76^c), d)^	3.68 ± 0.65^c)^	0.074
*P-*value	0.000	0.000	0.000	
mPI				
Baseline	1.81 ± 0.18	2.23 ± 0.53^a)^	2.12 ± 0.56	0.023
4th week	1.56 ± 0.21^c)^	1.35 ± 0.44^c)^	1.37 ± 0.39^c)^	0.202
12th week	1.48 ± 0.28^c)^	1.13 ± 0.33^a)^, ^c)^, ^d)^	1.06 ± 0.15^a)^, ^c), d)^	0.000
*P-*value	0.000	0.000	0.000	
GI				
Baseline	1.81 ± 0.23	2.04 ± 0.29	1.94 ± 0.27	0.078
4th week	1.50 ± 0.38^c)^	1.40 ± 0.42^a)^, ^c)^	1.10 ± 0.19^b)^, ^c)^	0.009
12th week	1.31 ± 0.38^c), d)^	1.10 ± 0.22^c), d)^	1.02 ± 0.07^a)^, ^c)^	0.022
*P-*value	0.000	0.000	0.000	

PD, CAL; Probing depth, Clinical attachment level

a), significant differences from group 1

b), significant differences from group 2

c), significant differences from baseline

d), significant differences from 4th week

**Table 3:** Changes in biochemical parameters over time

	Groups			
Parameters & Time	Mechanical Debridement	Diode Laser	Combined	*P* value
PISF				
Baseline	1.33 ± 0.65	1.16 ± 0.62	1.33 ± 0.55	0.672
4th week	1.20 ± 0.57	0.97 ± 0.66 ^a)^	0.87 ± 0.50 ^a)^	0.345
12th week	0.79 ± 0.38^a), b)^	0.55 ± 0.43^a), b)^	0.56 ± 0.34^a), b)^	0.256
*P value*	0.000	0.000	0.000	
S100A8 (ng)				
Baseline	4.51 ± 0.52	4.78 ± 0.37	4.63 ± 0.47	0.189
4th week	4.29 ± 0.44	4.63 ± 0.34	4.46 ± 0.45	0.134
12th week	4.41 ± 0.37	4.59 ± 0.36	4.38 ± 0.24	0.352
*P value*	0,872	0,133	0,689	

PISF; Peri−implant sulcus fluid

a), significant differences from baseline

b), significant differences from 4th week

### Baseline (T0) findings

In the baseline values, the groups showed similar distributions for PISF volume, S100A8 total amount, and GI variables, and no statistically significant differences were found (*p* > 0.05). However, statistically significant differences were found between the groups in baseline PD, CAL, and mPI (*p* < 0.05). The highest values for these parameters were observed in the diode laser group, and the lowest were observed in the mechanical debridement group.

### 4th week (T1) findings

At the 4th week after treatment, significant differences were found between the groups in CAL and GI values (*p* = 0.010 and *p* = 0.009, respectively). The highest CAL value was determined in the diode laser group, and the lowest value was determined in the mechanical debridement group. The highest GI value was observed in the mechanical debridement group, and the lowest value was observed in the combined treatment group. During this period, no statistically significant differences were found between the groups in PISF volume, S100A8 total amount, PD, and mPI variables (*p* > 0.05).

### 12th Week (T2) findings

At the end of the study, a significant difference persisted between the groups in mPI and GI values (*p* < 0.001). The highest values for both parameters were observed in the mechanical debridement group, and the lowest were observed in the combined group that received both mechanical debridement and a diode laser. Apart from this, no statistically significant differences were found between the groups in PISF volume, S100A8 total amount, PD, and CAL variables at the 12th week (*p* > 0.05).

### Changes over time and treatment efficacy (intra-group evaluation)

When treatment efficacy was evaluated over time, significant improvements in clinical parameters were observed across all study groups. PD showed a statistically significant decrease over time in all groups (*p* < 0.05). When comparing baseline and 12th week values, the most significant decrease in PD levels was observed in the combined treatment group. CAL showed a significant improvement over time in all groups, similar to PD (*p* < 0.05). Considering pre- and post-treatment values, the greatest decrease in CAL levels was observed in the diode-laser-treated group. A statistically significant decrease in mPI and GI over time was observed in all groups (*p* < 0.05). When the difference between the initial and final values was examined, the greatest decrease in both parameters was observed in the groups that received combined treatment. While PISF levels decreased significantly over time across all groups, the largest decrease was observed in the combined group. However, unlike clinical improvement, no statistically significant change in S100A8 total levels was detected over time across groups (*p* > 0.05).

## Discussion

The main objective of this study is to evaluate the effectiveness of combined therapies, applied as alternatives or additions to mechanical debridement, on clinical parameters and S100A8 levels in peri-implant sulcus fluid. Our study found that all three treatment protocols resulted in significant improvement in clinical parameters during the 12-week follow-up period. However, in the intergroup comparison, the combined treatment stood out as statistically superior in improving inflammatory parameters, such as mPI and GI, and as the most significant method for numerically reducing PD. In contrast, while the volume of PISF decreased in parallel with clinical improvement, no significant change in the total amount of S100A8, a neutrophil activation marker, was observed between groups or over time. Based on the findings of this study, the null hypothesis is rejected, as the combined treatment protocol provided significantly greater improvement in both clinical parameters and biochemical markers than monotherapies. The lack of a single, recognized gold-standard protocol for the treatment of peri-implantitis has increased the search for combined treatments [18]. The superior clinical results of the combined group in our study can be attributed to the synergy between mechanical debridement’s ability to physically remove biofilm and the diode laser’s bactericidal and photobiomodulatory effects. In the literature, Mettraux et al. (2016) and Tenore et al. (2020) similarly reported that diode laser treatment compared with mechanical debridement alone resulted in a greater reduction in PD and bleeding scores [19, 20]. The laser’s ability to eliminate bacteria in implant grooves and microporosities that mechanical instruments cannot reach, and its biostimulatory effect that accelerates soft tissue healing, are the basis of the clinical success in this study. In particular, the fact that the most significant gain in CAL was observed in the laser adjunctive groups in our study is consistent with the laser’s effect of increasing fibroblast proliferation and collagen synthesis [21, 22].

The most striking and debatable finding of our study is that, despite significant improvements in clinical parameters, S100A8 levels did not change significantly. S100A8, also known as calprotectin, is a marker released from neutrophils that typically peaks in the early stages of acute inflammation [23]. Although de Mello-Neto et al. (2021) reported a decrease in S100A8 levels at the 3rd month after peri-implantitis treatment, the absence of this decrease in our study can be explained by several mechanisms [13]. First, there is the timing of sampling. Acute-phase reactants, such as S100A8, may rapidly decrease and plateau in the first days or weeks after treatment. 12 week period is a chronic phase in which tissue remodeling begins; during this period, S100A8 levels may have returned to basal levels but may not have shown a sufficiently large change to reach statistical significance. Second, there is subclinical inflammation. Even if the gingiva appears clinically healthy, neutrophil activity may remain low at the histological level. Altindal et al. (2023) similarly reported that although clinical improvement occurred after laser treatment, biomarkers such as IL-1ß did not decrease [24]. This suggests that the laser rapidly suppresses clinical symptoms, whereas the immune response at the molecular level is a more complex, long-term process.

In our study, 10% povidone iodide was used to provide standard decontamination in all groups. Sahrmann et al. (2010) reported that adding povidone-iodine to mechanical debridement provided an additional benefit in clinical pocket depth. One reason for the clinical improvement observed across all groups in this study may be that povidone-iodide reduces the effective microbial load. However, the difference between the groups favoring the laser confirms the laser’s specific effects on the tissue rather than those of the chemical agent [25].

Some limitations of our study should be considered. First, although the 12-week follow-up period is sufficient to evaluate the early inflammatory response and the clinical improvement from treatment, it is limited in drawing definitive conclusions about the long-term stability of the gains achieved. Secondly, our study specifically focused on the diagnostic value of S100A8; future studies including other markers of bone metabolism and inflammation, such as osteocalcin or TNF-α, could provide a broader molecular picture of peri-implant tissue degradation. Finally, the results of this study should be supported by multicenter studies with larger sample sizes and longer follow-up periods.

In conclusion, this study has shown that the combined use of mechanical debridement and a diode laser in the treatment of peri-implantitis provides more effective clinical results than methods applied alone. The fact that S100A8 levels did not change despite clinical improvement indicates that clinical parameters and biochemical markers may not always move in step during the follow-up of peri-implant diseases; therefore, clinical examination remains the most reliable method for evaluating treatment success.

## Data Availability

The datasets generated during and/or analyzed during the current study are available from the corresponding author on reasonable request.
